# Integration of Genome-Wide DNA Methylation and Transcription Uncovered Aberrant Methylation-Regulated Genes and Pathways in the Peripheral Blood Mononuclear Cells of Systemic Sclerosis

**DOI:** 10.1155/2018/7342472

**Published:** 2018-09-02

**Authors:** Honglin Zhu, Chengsong Zhu, Wentao Mi, Tao Chen, Hongjun Zhao, Xiaoxia Zuo, Hui Luo, Quan-Zhen Li

**Affiliations:** ^1^Department of Rheumatology & Immunology, Xiangya Hospital, Central South University, 87 Xiangya Road, Changsha, Hunan 410008, China; ^2^Department of Immunology and Internal Medicine, University of Texas Southwestern Medical Center, 5323 Harry Hines Blvd., Dallas, TX 75390, USA

## Abstract

*Objective. *Systemic sclerosis (SSc) is a systemic connective tissue disease of unknown etiology. Aberrant gene expression and epigenetic modifications in circulating immune cells have been implicated in the pathogenesis of SSc. This study is to delineate the interaction network between gene transcription and DNA methylation in PBMC of SSc patients and to identify methylation-regulated genes which are involved in the pathogenesis of SSc.* Methods. *Genome-wide mRNA transcription and global DNA methylation analysis were performed on PBMC from 18 SSc patients and 19 matched normal controls (NC) using Illumina BeadChips. Differentially expressed genes (DEGs) and differentially methylated positions (DMPs) were integrative analyzed to identify methylation-regulated genes and associated molecular pathways*. Results.* Transcriptome analysis distinguished 453 DEGs (269 up- and 184 downregulated) in SSc from NC. Global DNA methylation analysis identified 925 DMPs located on 618 genes. Integration of the two lists revealed only 20 DEGs which harbor inversely correlated DMPs, including 12 upregulated (ELANE, CTSG, LTBR, C3AR1, CSTA, SPI1, ODF3B, SAMD4A, PLAUR, NFE2, ZYX, and CTSZ) and eight downregulated genes (RUNX3, PRF1, PRKCH, PAG1, RASSF5, FYN, CXCR6, and F2R). These potential methylation-regulated DEGs (MeDEGs) are enriched in the pathways related to immune cell migration, proliferation, activation, and inflammation activities. Using a machine learning algorism, we identified six out of the 20 MeDEGs, including F2R, CXCR6, FYN, LTBR, CTSG, and ELANE, which distinguished SSc from NC with 100% accuracy. Four genes (F2R, FYN, PAG1, and PRKCH) differentially expressed in SSc with interstitial lung disease (ILD) compared to SSc without ILD.* Conclusion.* The identified MeDEGs may represent novel candidate factors which lead to the abnormal activation of immune regulatory pathways in the pathogenesis of SSc. They may also be used as diagnostic biomarkers for SSc and clinical complications.

## 1. Introduction

Systemic sclerosis (SSc) is a chronic autoimmune disease characterized by microvascular dysfunction, immune abnormalities, chronic inflammation, and interstitial and perivascular fibrosis in the skin and internal organs [1]. Pulmonary arterial hypertension (PAH) and interstitial lung disease (ILD) are the most common pulmonary complications in SSc, and PAH is the leading cause of mortality in SSc patients [2]. SSc is a clinically heterogeneous disease with unknown etiopathogenesis and presents with distinct subphenotypes [3]. The exact cellular and molecular mechanism of SSc remains unclear.

Considerable evidences from genome-wide association studies (GWAS) support the inheritable nature of the disease by the identification of susceptibility genes that have been attributed to immune regulation, inflammation, transcription, kinase activity, DNA cleavage, and repair in SSc [4–6]. In addition, the environmental influence via epigenetic mechanisms, particularly altered status of DNA methylation, contributes to the environment-host interaction in the development of SSc [7, 8]. DNA methylation profiling in SSc has been obtained by Illumina methylation arrays, which identifies genes with differential methylation. It has been reported that more than 60 methylation-regulated genes were associated with autoimmunity in SSc [5, 9, 10]. However, there were no studies that investigate the correlation between gene expression and DNA methylation at global genome level in SSc. The advances in genome-wide technologies have provided unprecedented opportunities to expand our view of the relationship among the genome, methylome, and transcriptome. The integration of epigenetic and genetic data promises to gain insight into the mechanisms affecting epigenetic alteration, gene expression, and disease susceptibility [11, 12].

SSc is a systemic disease and involves multiple organs, tissues, and cell types. Previous data were generated from fibroblast [13, 14], purified CD4^+^ T cells [8, 14, 15], whole blood cells [16], and skin biopsy [17]. Peripheral blood mononuclear cells (PBMCs) represent a broad spectrum of cell types including T cells, B cells, NK cells, monocytes, and dendritic cells, which all played major roles in immunological events. Its ready accessibility, in particular in repeat sampling for serial comparison of profiling, has made PBMC an ideal target in obtaining a comprehensive picture of immune status.

In this study, we performed a comprehensive screening for global patterns of aberrant DNA methylation and gene expression in the PBMC of SSc and healthy control in order to identify methylation-regulated genes and the enriched pathways and to understand the functional consequence of DNA methylation aberrancy on the regulation of gene expression in SSc.

## 2. Materials and Methods

### 2.1. SSc Patients and Controls

18 SSc patients (14 diffuse cutaneous and 4 limited cutaneous SSc) and 19 normal controls enrolled in the study in the Department of Rheumatology, Xiangya Hospital, Central South University, Changsha, China. All patients and controls are Han Chinese. The diagnosis of all patients meets the ACR classification criteria for SSc [18]. The clinical features of SSc patients included in this study are shown in Table 1. ILD was diagnosed with high-resolution computed tomography (HRCT) when ground-glass opacity or reticulation was detected in nondependent portions of lung or ground-glass opacity and reticulation was found in dependent portions of lung that persisted on prone imaging and the presence of honeycombing and traction bronchiectasis [19–21]. The gastrointestinal involvement was evaluated by GI symptoms and nutrition status, including reflux, bloating, distension, constipation, diarrhoea, anorectal incontinence, weight loss, and malnutrition [22]. The institutional review board at Xiangya Hospital, Central South University, approved this study. All study participants signed a written informed consent prior to participation.

### 2.2. DNA and RNA Isolation

Peripheral blood samples were obtained from SSc patients and normal controls. The PBMC were separated from heparinized blood by density gradient centrifugation over Ficoll-Hypaque plus (GE Healthcare, Piscataway, NJ, USA). Total RNA was isolated from PBMC by standard phenol-chloroform extraction using Trizol reagent (Invitrogen Life Technologies, Carlsbad, CA) according to the manufacturer's instructions. Genomic DNA was isolated from whole blood cells using genomic DNA extraction kits (Life Technologies, Gaithersburg, MD).

### 2.3. mRNA Transcription Profiling

The genome-wide mRNA transcriptome in PBMC from 18 SSc patients and 19 NC was analyzed using Illumina humanHT-12 v4.0 BeadChip arrays bearing 48,700 human gene transcripts following vendor's instruction. Briefly, 500 ng total RNA from each sample was used to generate biotin-labeled cRNA probe which was then hybridized onto genechip array. After staining with Cy3-labeled streptavidin (Amersham, Piscataway, NJ, USA), slide was scanned on an Illumina HiScan scanner and the image was analyzed using GenomeStudio v3 software (Illumina, Inc.) to generate bead-summarized data. Further analysis on the gene expression data was performed using lumi package in R [48]. The ReMOAT annotation of gene expression data was used to include only ‘‘perfect” and ‘‘good” annotated probes [49]. Exploratory quality-control analyses revealed no strong batch effects. After quality control and preprocessing of Illumina arrays, differential gene expression between disease and normal group was performed using limma package in R and multiple comparisons correction was performed in R [50]. For genes with multiple transcripts, we selected the transcript with the highest fold change between SSc and normal. The differentially expression genes were called using our criteria of BH- (Benjamini-Hochberg-) adjusted* p* < 0.05 and absolute fold change >1.5.

### 2.4. Genome-Wide DNA Methylation Analysis

DNA methylation status of 485,000 CpG sites across the entire genome was interrogated using the Illumina Human Methylation 450K BeadChip arrays (Illumina, San Diego, CA). This methylation array covers 99% of RefSeq genes, with an average of 17 CpG sites per gene across the promoter region, 50 sites in untranslated region (5'-UTR), first exon, gene body, and 3'-UTR. It covers 96% of CpG islands, non-CpG-islands methylation sites, and microRNA promoter regions. Genomic DNA (1*μ*g) extracted from PBMC was bisulfite converted using EZ DNA Methylation kit (Zymo Research Corp, Orange, CA), and a cyclic denaturation step was used during the conversion reaction. Whole-genome amplification, fragmentation, resuspension, hybridization, washing, extending and staining, and scanning were performed according to the manufacturer's instructions. The arrays were scanned using Illumina HiScan scanner and generated the raw IDAT format files which were analyzed using the Chip Analysis Methylation Pipeline (ChAMP) implemented in R and available from Bioconductor [51]. Briefly, raw IDAT files were used as input data and probes were filtered by their raw intensity values using a detection* p*-value threshold of 0.01. Probes corresponding to the X and Y chromosomes were removed from the dataset as both male and female samples were being analyzed. Locus-by-locus analyses were conducted using the nonparametric Wilcoxon rank-sum test, and multiple comparisons correction was performed in R [50]. A CpG site was considered statistically differentially methylated only if the BH-adjusted* p* value < 0.05 between the tested groups and the absolute difference of the median *β*-value is greater than 0.12. For genes with multiple probes measuring DNA methylation, we selected the probe with the highest fold change value for DNA methylation.

### 2.5. Real-Time PCR Validation of the DEGs

DEGs were validated using Taqman assays on a 7900HT Fast Real-Time PCR system (Applied Biosystems, Foster City, CA, USA). The assay was performed by using a TaqMan RNA-to-CT 1-Step kit (Applied Biosystems) in a total volume of 20 *μ*l, which contained a final concentration of 900 nM sense and antisense primers (Supplementary Table 1), 250 nM Taqman gene probe, 1× TaqMan RT Enzyme Mix, and 1 × TaqMan RT-PCR Mix. The cDNA amplification was monitored using 7900HT Fast Real-Time PCR system under the conditions of 48°C for 15 min, 95°C for 10 min, and 40 cycles of 95°C for 15 s and 60°C for 1 min. This assay was carried out in triplicate for each sample, including a no-template control. The relative quantity (RQ) of the gene expression in each sample was calculated by normalizing to housekeeping gene GAPDH.

### 2.6. Integrative Analysis of Transcriptome and DNA Methylation

Each methylation probe was mapped to the nearest transcript starting site. Transcription information of hg19 was fetched from UCSC Genome browser database and further processed using the Bioconductor Genomic Feature package. A probe was mapped to the nearest gene if the distance between the probe and the nearest gene's transcription starting site was less than 10 kilobases. We retained only the subset of probes associated with genes that were represented on the gene expression microarray. This resulted in the retention of 16,750 genes associated with the CpG probes in methylation and transcription probes in gene expression. Pearson correlation coefficients for each annotated gene were first calculated among all possible pairs of methylation probe sets and gene expression probe sets between SSc and normal control group. The methylation-expression probe set pair with the maximum absolute correlation coefficient was then chosen for each gene.

### 2.7. Support Vector Machines

Support vector machines (SVMs) are supervised learning models traditionally employed for classification analysis through constructing a model based on a separating plane that maximizes the margin between different classes [52]. A set of differential expression genes (DEGs) will be used to evaluate whether selected DEGs will correctly classify normal control from SSc patient samples. We begin by choosing radial kernel and tune the optimal model parameters (i.e., cost and gamma) to achieve the best diagonal performance on hold-on-one-out cross-validation test [53]. The SVM is trained using data from all but one of the sample. The sample not used in training is then assigned a class by the SVM. A single SVM experiment consists of a series of hold-one-out experiments, each sample being held out and tested exactly once. The e1071 R package is used for implement the SVM analysis.

### 2.8. Bioinformatics Analysis

Differentially methylated and/or expressed genes were analyzed for Gene Ontology (GO) enrichments in comparison to all genes available on the Illumina Infinium Human Methylation 450K platform using the DAVID Functional Annotation Tool [54] Genes for which expression levels change in concordance with DNA methylation changes were analyzed for gene network and biological processes enrichment using IPA (Ingenuity Pathway Analysis: http://www.qiagenbioinformatics.com/products/ingenuity-pathway-analysis). Meanwhile, GAGE package in R [55] was used for detection of gene enrichment in KEGG pathway and GO analysis; the enriched pathway was visualized by Pathview package in R [56].

## 3. Results

### 3.1. Identification of DEGs in PBMC of SSc

**Figure 1:**
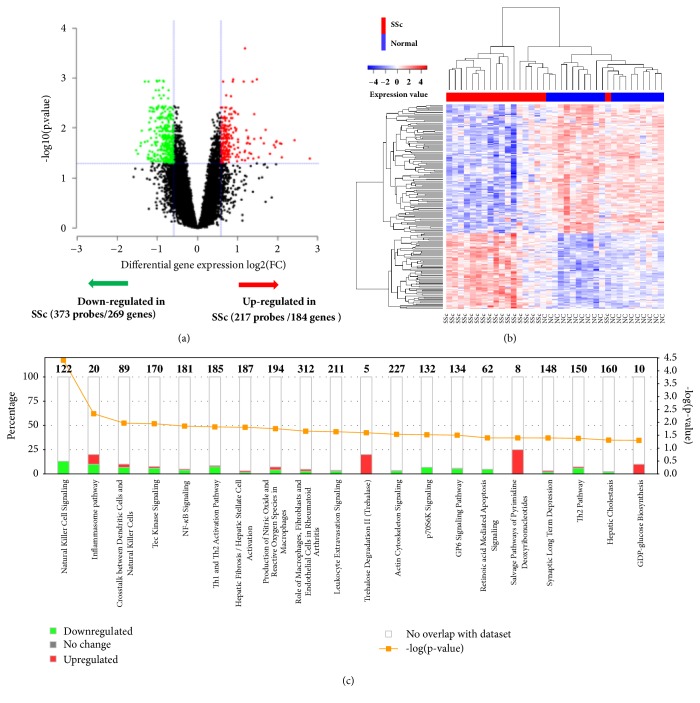
**Identification of differentially expressed genes between systemic sclerosis and normal.** (a) Volcano plot of the differential gene expression analysis. X-axis: fold change difference (log 2 scale); y-axis: BH-adjusted* p* values for each probe (- log10 scale). The vertical dotted lines represent absolute cutoff value of 1.5-fold change. The horizontal dotted line represents the significant cutoff of* p* < 0.05 (see Supplementary Table 2). (b) Two-dimensional hierarchical clustering of differential gene expression probes. Probes are shown in rows; samples are shown in columns. (c) Canonical pathway analysis of the differentially expressed genes in PBMC from SSc patients as compared with normal controls. Statistically significant pathways were shown. The 1^st^ y-axis indicates percentage of differential expression genes involved in the pathway. The 2^nd^ y-axis shows BH-adjusted* p*-value of enrichment analysis.

We performed a genome-wide probe-based differential gene expression analysis using 47,312 probes after QC procedure to identify differentially expression probes including covariates for age, sex, and the first two principal components from expression values of all the probes, which is routinely included in GWAS analyses to control for population stratification [57]. By comparison of the whole-genome transcription profile of PBMC between patients with SSc and NCs, we identified 590 differentially expressed transcripts (Supplementary Table 2-1), representing 453 unique differentially expressed genes (DEGs) in SSc, which include 184 upregulated DEGs and 269 downregulated DEGs (Figure 1(a); Supplementary Table 2-2). Hierarchical clustering of the DEGs demonstrated that the patterns of expression profiles clearly distinguished NC from SSc patients with the exception that four SSc were misclassified in the NC group (Figure 1(b); Supplementary Table 2). Among the upregulated DEGs, 10 DEGs belong to the family of IFN-inducible genes, 6 DEGs are complement components, and 3 DEGs are *α*-defensins (Table 2). Induction of interferon signature genes and complement activation were reported in a number of autoimmune diseases including SSc and its complications [58–61]. Human *α*-defensins encode human neutrophil peptides (HNPs) and function as enhancer of phagocytosis by macrophages. Defensins were associated with mucosal immunity [62] and were implicated in the pathogenesis of ILD in SSc [63]. By examining the downregulated DEGs, we found 19 genes are associated with NK cells (Table 2), implying a compromised NK cell function. The DEG profiling identified in our study highlighted a crucial contribution of innate immunity in the pathogenesis of SSc.

Pathway analysis using Ingenuity Pathway Analysis software (IPA) indicated that the 453 DEGs are most significantly involved in the pathways of NK cell signaling, inflammasome, crosstalk between dendritic cells and NK cells, Tec kinase signaling, and NF-*κ*B signaling (Figure 1(c)). Among the 184 overexpressed DEGs, 48 genes are associated with rheumatic diseases, 40 genes are involved in viral infection, and 34 genes are related to inflammatory response (Supplementary Figure 1).

DEGs were also analyzed for GO enrichments using the DAVID Functional Annotation Tool [54]. GO analysis demonstrated that the DEGs were significantly enriched in “immune system process”, “immune response”, and “regulation of immune system process”. Additionally, GO for downregulated genes were enriched in “NK cell mediated immunity” (Supplementary Figure 2).

### 3.2. Identification of Differentially Methylated Positions (DMPs) in SSc

**Figure 2:**
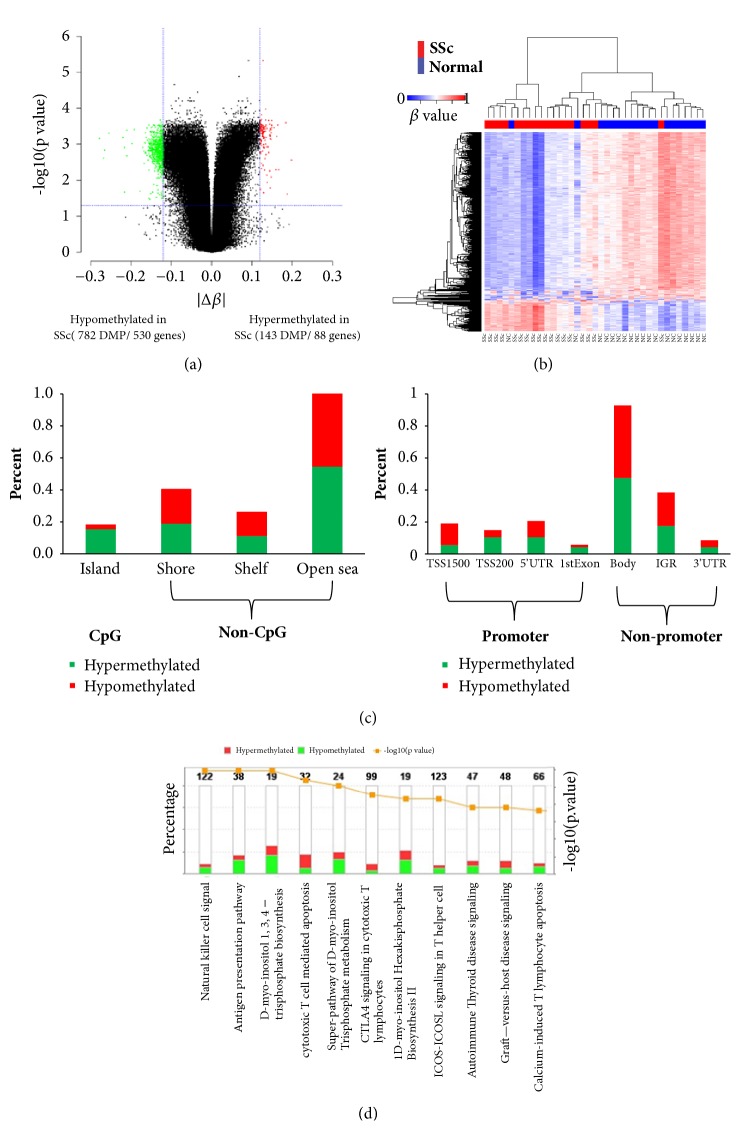
**Identification of DNA methylation differences between systemic sclerosis and normal.** (a) Volcano plot of the differential DNA methylation analysis. X-axis: mean *β*-value difference; y-axis: BH-adjusted* p* values for each probe (- log10 scale). The vertical dotted lines represent 12% change in *β*-values. The horizontal dotted line represents the significant cutoff of* p* < 0.05. Seven genes, ANKFY1, FAM49B, GNG7, INPP5A, LAX, PITPNM2, and VOPP1, showed both significant hypermethylation and hypomethylation (see text and Supplementary Table 2). (b) Two-dimensional hierarchical clustering was performed using the differential Infinium DNA methylation probes across all samples (n = 37). Probes are in rows; samples are in columns. (c) Proportions of probes from genes with associated CpG islands, shelf, shore, and open sea (left) and proportions of probes from genes with associated probe locations, categorized as gene body, intergenic, 3'UTR, 5'UTR, 1^st^ exon, TSS1500, and TSS200 (right). (d) Canonical pathway analysis of the differentially expressed genes in PBMC from SSc patients as compared with normal controls. Statistically significant pathways were shown. The 1^st^ y-axis indicates percentage of differential expression genes involved in the pathway. The 2^nd^ y-axis shows BH-adjusted p-value of enrichment analysis.

By performing a locus-by-locus differential DNA methylation analysis with age, sex, and the first two principal components as covariates, we identified a total of 925 differential methylated positions (DMPs) in SSc patients, among which 782 DMPs (85%) were hypomethylated and 143 DMPs (15%) were hypermethylated (Figure 2(a); Supplementary Table 3). The two-dimensional hierarchical clustering with DMGs resulted in separation of SSc from normal controls, with only minimal number of misclassified samples (one NC and two SSc) (Figure 2(b)).

Next, we investigated the genome distribution of the 925 DMPs in PBMC of SSc. Surprisingly, only 5% (45 of 925) of DMPs were located on CpG islands and the rest were found in non-CpG islands, mostly in open sea regions, followed by shore and shelf (Figure 2(c) and Supplementary Table 4). Based on the position of the DMPs, we found that about 30% DMPs were located in the promoter regions, including TSS1500, TSS200, 5'UTR, and the first exon, whereas the majority of DMPs were found within nonpromoter regions, mostly in gene body, followed by IGR and 3'UTR. Hypomethylated or hypermethylated DMPs demonstrated a very comparable genomic distribution proportion within CpG island/non-CpG island and promoter/nonpromoter regions (Figure 2(c) and Supplementary Table 3).

The 925 DMPs were mapped onto 618 unique genes and defined as differentially methylated genes (DMGs). Some of the DMGs have been previously reported in SSc and other autoimmune diseases [64]. Several interferon-inducible genes, including PAPP9, MX1, IFI44L and PLSCR1A, were among the top hypomethylated genes. PF4 and HLA-C were top hypermethylated genes. PF4 is a marker for activation of endothelial cell and coagulation that was implicated in vasculopathy of SSc [65–67]. HLA-C is coexpressed with certain killer cell immunoglobulin-like receptors (KIRs) in SSc and SLE [68] and may involve in the innate immunity.

Pathway analysis of the 618 DMGs indicated the DMGs were enriched in natural killer (NK) cell signaling, antigen presentation, D-myo-inositol 1, 3, 4-trisphosphate biosynthesis, and cytotoxic T cell mediated apoptosis (Figure 2(d)).

GO analysis revealed that the differentially hypomethylated genes were significantly enriched in the processes of regulation including alternative splicing, phosphoprotein, and polymorphism, which may result in modification of transcription and translation by DNA methylation aberrancy, whereas the differentially hypermethylated genes were considerably enriched in adaptive immunity and intracellular signal transduction (BH-adjusted* p* < 0.05) (Supplementary Figure 3).

### 3.3. Identification of DEGs Associated with Altered DNA Methylation

**Figure 3:**
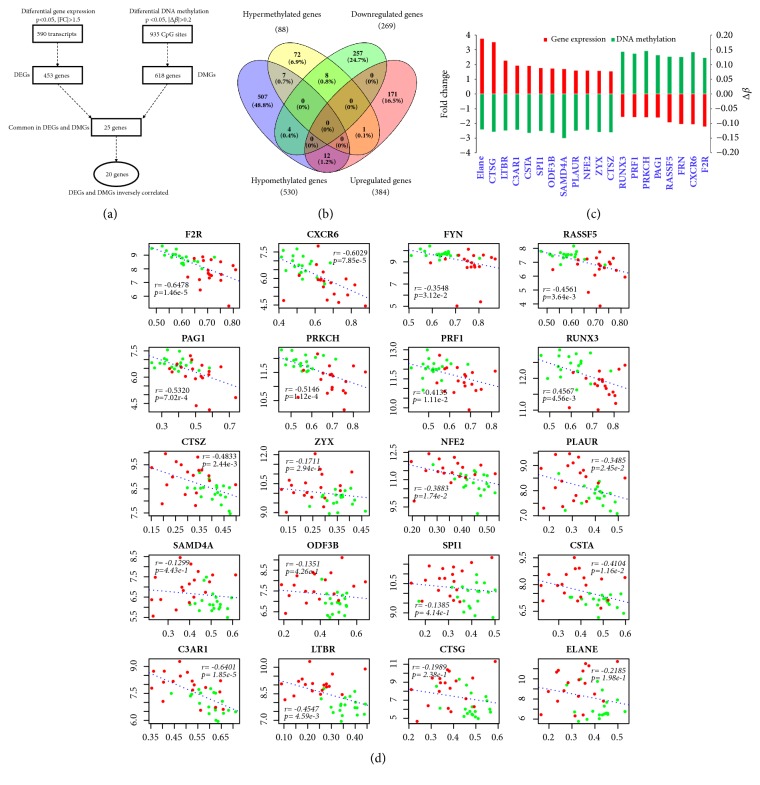
**The genes that shows the most significant changes in DNA methylation and gene expression.** (a) Flowchart of identification of genes showing coordinately changed DNA methylation and gene expression. (b) Venn diagram of differential DNA methylation and differential gene expression analysis. (c) The top-rank relevant differentially methylated sites with different gene expression in SSc compared with controls. The 1^st^ y-axis represents fold change of gene expression between SSc and normal control. The 2^nd^ y-axis represents Δ*β* of DNA methylation between SSc and normal control. (d) Correlation plots of DNA methylation versus gene expression in SScs and normal for the most significant changes in DNA methylation and gene expression.

To assess the functional relevance between the changes on gene expression and associated DNA methylation in PBMC of SSc patients, we integrated the gene expression profiles with DNA methylation profiles by Entrez gene ID. By integration of the 453 DEGs and 618 DMGs, we only identified 25 common genes which showed significant changes on their gene expression level (*p *< 0.05, |FC| > 1.5) and also contained at least one significantly altered DNA methylation site (*p *< 0.05, |Δ*β*| > 0.12) on the gene (Figure 3(a)). Among the 25 methylation associated genes, 20 genes demonstrated an inverse correlation on the changes between gene expression and DNA methylation (Figures 3(b)–3(d); Table 3), including 12 upregulated genes that were hypomethylated, and eight downregulated genes that were hypermethylated (Figures 3(c)-3(d)). The negative correlations between gene expression and DNA methylation indicate that these genes are possibly methylation-regulated DEGs (MeDEGs) in the PBMC of SSc.

To further define the regulatory effect of the DNA methylation sites on gene expression, we investigated the location of each DMP in the genomic regions of the 20 genes. We found the DMPs were located in the promoter regions of 12 genes (60%), including hypomethylated DMPs on seven upregulated genes (CSTA, CTSG, CTSZ, ELANE, LTBR, NFE2, and ODF3B) and hypermethylated DMPs on the five downregulated genes (CXCR6, FYN, PAG1, PRF1, and RUNX3). For the rest eight MeDEGs, the DMPs were found in the gene body (Supplementary Table 5). There is evidence that gene bodies are also involved in the regulation of gene expression [69, 70]. The genomic distribution of the DMPs suggested the DEGs may be methylation-regulated and yet to be confirmed. However, our data revealed only 4.4% of DEGs (20 of 453) harbored significant altered methylation sites, implying that DNA methylation may not play a major role on the gene expression profile in the PBMC of SSc.

In addition, five genes showed positive correlation between DNA methylation and gene expression changes. One gene, HLA-C, was hypermethylated and upregulated, and four genes, MBP, NKTR, GNG2 and SPTBN1, were hypomethylated and downregulated in SSc (Figure 3(b); Supplementary Table 5).

### 3.4. Validation of the Expression of MeDEGs by QPCR

**Figure 4:**
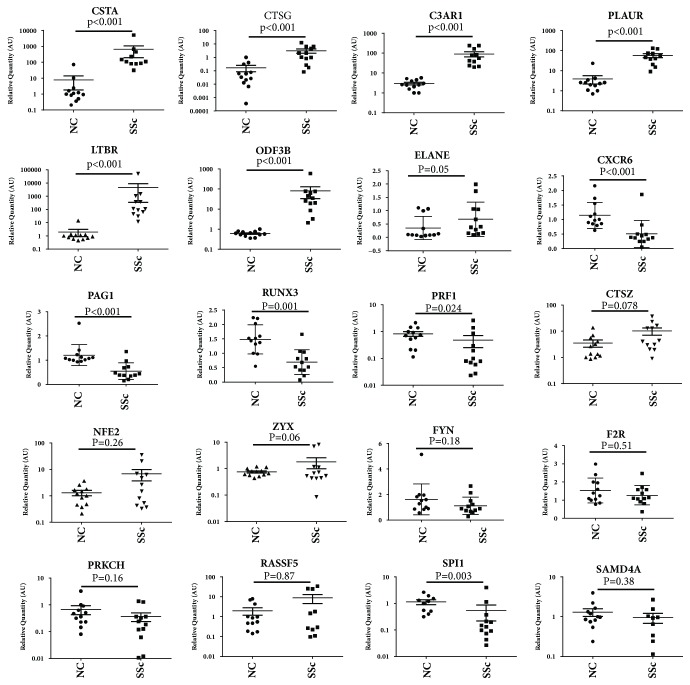
**QPCR validation of gene expression: 20 MeDEGs were validated by QPCR in a separate cohort of SSc (n=12) and NC (n=12) samples.** 11 MeDEGs (7 upregulated and 4 downregulated) were confirmed to be differentially expressed between SSc and NC (*p*<0.05) by QPCR and 6 MeDEGs (3 upregulated and 3 downregulated) showed the difference in expression but did not reached statistical significance. Inconsistency in the detection of the expression for three MeDEGs by microarray or QPCR was also noted.

The expression of the 20 MeDEGs was further validated by quantitative RT-PCR (QPCR) in a separate cohort of SSc (n=12) and NC (n=12). 11 MeDEGs were confirmed to be differentially expressed in SSc (*p*<0.05), including seven upregulated genes (CSTA, CTSG, C3AR1, PLAUR, LTBR, ODF3B, and ELANE) and four downregulated genes (CXCR6, PAG1, RUNX3, and PRF1) (Figure 4). Six MeDEGs showed an increasing trend (CTSZ, NFE2, and ZXY) or decreasing trend (FYN, F2R, and PRKCH), but the change was not statistically significant, possibly because of the small sample size. The overall consistency on the transcriptional level between the two assays by microarray and QPCR was observed in 17 of 20 MeDEGs. However, inconsistency was also noted in three genes (RASSF5, SPI1, and SMAD4A). Further confirmation is undergoing with an expanded sample collection.

Some MeDEGs have been reported as susceptibility genes in SSc, such as hypomethylated RUNX3 [13], which was in consistence with our result. Most of them were not associated with SSc and their role in SSc remains elusive. However, ample evidence indicated that the MeDEGs are involved in the characteristic pathology of SSc, including extracellular matrix remodeling (CSTA, CTSZ, CTSG, and ELANE), angiogenesis (CTSZ, CTSG, and CXCR6), coagulation/fibrolytic system (PLAUR and F2R), inflammatory response (C3AR1), and transcription factors associated with DEGs (NEF2, SPI1, and RUNX3) (Table 3).

### 3.5. Association of DNA Methylation and Gene Expression Profiling with Clinical Phenotype in SSc

**Figure 5:**
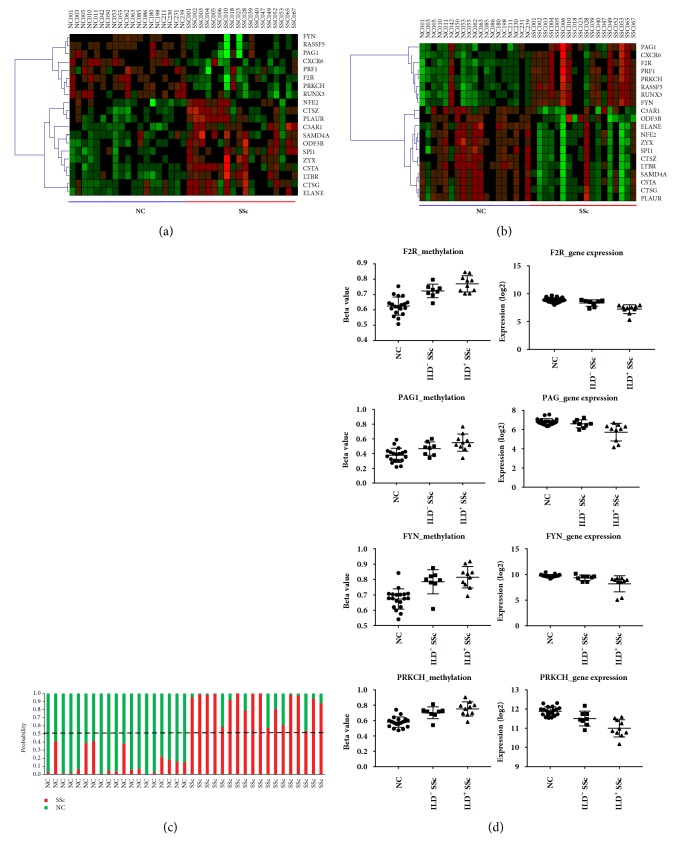
**Association of clinical characteristics of SSc and 20 DEGs. **(a) Heatmaps of gene expression and (b) DNA methylation show the most significant 20 correlated genes for SSc versus Normal. (c) Support vector machines analysis. The y-axis shows the group proportion inferred by SVM and sample ID is on the x-axis. (d) Array-based DNA methylation and mRNA expression among normal controls, the SSc with ILD, and SSc without ILD group,* p*-value < 0.05 between SSc with and without ILD groups, are showed.

Identification of 20 MeDEGs in SSc has enabled us to differentiate the disease status and/or stage of SSc. We performed an exploratory two-dimensional (2D) hierarchical clustering with the 20 MeDEGs across the 37 samples with the 20 MeDEGs. As expected, profiling with either DEGs (Figure 5(a)) or DMGs (Figure 5(b)) resulted in separate sample clusters, SSc and NCs, with a few exceptions. On the other hand, the misclustered samples may be associated with the pathogenic transition of disease status. In order to precisely differentiate SSc from NC, we applied SVM algorithm on the 20 MeDEGs. By fitting various combinations of the 20 MeDEGs, we further identified a set of six DEGs, i.e., F2R, CXCR6, FYN, LTBR, CTSG, and ELANE, that completely separate the SSc and NC population. All the 19 NCs are predicted as healthy controls, and the 18 SSc patients are all classified as SSc (Figure 5(c)). The six MeDEGs may be potentially used as diagnostic markers.

Diffuse and limited are two subsets of SSc, which are classified mainly by clinical features. To answer the question whether the MeDEGs may differentiate dsSSc from lcSSc, we assessed the expression for the 20 MeDEGs in the subsets. However, we did not find the difference in gene expression or methylation between dSSc and lSSc (data not shown). As we only have four lSSc and 14 dSSc, the assessment needs to be validated in an expanded population.

Interstitial lung disease (ILD) frequently complicates SSc and can be a deliberating disorder with poor prognosis. We then attempted to characterize a profiling that distinguishes ILD from non-ILD SSc patients. Initial analysis with locus-by-locus DMP or transcript-by-transcript DEG was not distinguishable between ILD and non-ILD SSc patients (data not shown). We then reexamined the level of mRNA expression and DNA methylation of the 20 MeDEGs in NC, ILD, and non-ILD SSc patients. The level of mRNA expression and DNA methylation was evaluated across three groups. The expression for four underexpressed MeDEGs, F2R, FYN, PAG1, and PRKCH, was significantly reduced in SSc with ILD as compared to SSc without ILD (*p* <0.05) (Figure 5(d)). The corresponding methylation level for the four genes tended to be increasing, but the difference was not significant. The finding can be interpreted as a correlation between downregulated expression for F2R, FYN, PAG1, and PRKCH and the development of ILD in SSc. Further, these four MeDEGs may be used as diagnostic or prognostic markers for SSc with pulmonary involvement.

Serum Scl-70 antibodies are the hallmark of systemic sclerosis and are found in up to 60% of patients with this connective tissue disease. Scl-70 antibodies are more common in patients with extensive cutaneous involvement and interstitial pulmonary fibrosis and are considered as a poor prognostic sign. Likewise, characterization of a gene profile in patients with positive Scl-70 antibody with the 20 MeDEGs was attempted but failed to identify a distinguishable gene set (data not shown).

### 3.6. Gene Networks Affected by 20 MeDEGs

**Figure 6:**
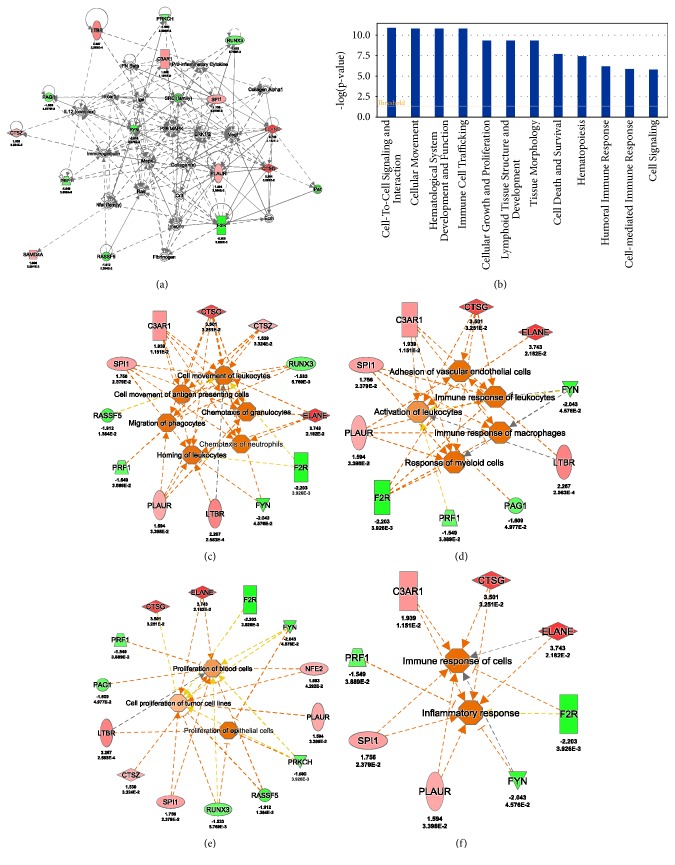
**The diseases and functions analysis by integrated pathway analysis using 20 MeDEGs.** (a) The top interaction networks involved by 15 MeDEGs. (b) Canonical pathway analysis of 20 MeDEGs in PBMC from SSc patients as compared with normal controls. Statistically significant pathways were shown. (c) Most genes (12/20) are involved with cell movements of leukocytes, cell movement of antigen presenting cells, migration of phagocytes, chemotaxis of granulocytes, homing of leukocytes, and chemotaxis of neutrophils. (d) The network from 10 DEGs is involved with adhesion of vascular endothelial cells, immune response of leukocytes, activation of leukocytes, immune response of macrophages, and response of myeloid cells. (e) Most genes (12/20) are involved with proliferation of blood cells, cell proliferation of tumor cell lines, and proliferation of epithelial cells. (f) The network from 8 genes is involved with immune response of cells and inflammatory response.

Finally, we explored the function connection and molecular interaction networks associated with the 20 MeDEGs using pathway analysis tools. A total of 4 interaction networks were identified (Table 4). The highest scoring (score = 41) molecular interaction network is related to cellular movement and immune cell trafficking which involved 15 of the 20 MeDEGs (Figure 6(a)). The MeDEGs are most significantly enriched in the molecular pathways associated with cell-to-cell signaling and interaction (*p*=1.26x10^−11^), cellular movement (*p*=1.56x10^−11^), hematological system development and function (*p*=1.56x10^−11^), immune cell trafficking (*p*=1.56x10^−11^), and cellular growth and proliferation (*p*=4.6x10^−10^) (Figure 6(b)). Most of the MeDEGs are connected with cell migration, trafficking and chemotaxis (11 genes, Figure 6(c)), endothelial adhesion and immune cell activation (10 genes, Figure 6(d)), proliferation of immune cells (14 genes, Figure 6(e)), and inflammatory and immune response (8 genes, Figure 6(f)). The results indicated that the MeDEGs play a pivotal role in the cascades of inflammatory response by orchestrating such a sequential process of inflammation: initiation, activation, and amplification of immune response.

## 4. Discussion

By comparison of the whole-genome transcriptome and DNA methylation in the PBMC of SSc and NC, we identified 453 DEGs and 618 DMGs which were significantly altered in SSc. However, integration of the DEGs and DMGs only derived 20 genes in which the gene expression is inversely correlated with the DNA methylation and is potential methylation-regulated DEGs (MeDEGs). To our knowledge, this is the first study to comprehensively investigate the effect of methylation on the gene transcription as the etiology of SSc at global genome level. The advantage of the integrative analysis lies in the simultaneous examination of the status or level change for both transcriptome and methylome which elucidated an immediate, real-time relationship between gene expression and DNA methylation. With the integrative analysis strategy, we have previously identified a large number of methylation-regulated genes in the PBMC of SLE patients [71]. In this study, we applied a more stringent criterion for identification of DEGs (*p* < 0.05, |FC| > 1.5) and DMGs (*p* < 0.05, |Δ*β*| > 0.12) in the PBMC of SSc patients, and only 25 genes were overlapped between the two lists, in which 20 DEGs were inversely correlated with aberrant DNA methylation. The difference in methylation frequency in SLE and SSc may reflect a different underlying mechanism in disease spectrum.

We then investigated the 20 MeDEGs for their potential role in the pathogenesis of SSc. Pathway analysis revealed that these MeDEGs are significantly enriched in the molecular pathways related with cell-to-cell signaling and interaction, cell migration, immune cell trafficking, hematological system development and function, and cellular growth and proliferation. The dysregulation of these genes may promote the migration of immune cells (macrophage, leukocytes, and neutrophils) to the pathogenic tissues, activation, and proliferation of immune cells and may eventually lead to local pathogenesis. Some of the gene may also induce adhesion and proliferation of vascular endothelial cells which can cause abnormal blood vessel growth [72]. Among the upregulated MeDEGs, ELANE, CTSG, and CTSZ are three proteases involved in remodeling of extracellular matrix. ELANE which encodes neutrophil elastase, together with matrix metalloproteinase 12 (MMP12), has been reported in the development cystic fibrosis in lungs [73]. CTSG and CTSZ are cathepsin proteases produced by macrophages. Cathepsin G leads to destruction of the lung matrix and continued propagation of acute inflammation [28]. Cathepsin Z promotes tumor invasion by interacting with integrins and the extracellular matrix [74]. The role of these genes in SSc has yet to be defined, but we speculated that overexpression of ELANE, CTSZ, and CTSG may involve dysregulation of extracellular matrix and subsequent fibrosis. Fibrosis in SSc is preceded by vascular injury, which in turn leads to tissue hypoxia and capillary rarefaction. Vascular injury potentially activates coagulation cascade and production of thrombin, which may trigger myofibroblast differentiation.

Urokinase-type Plasminogen Activator (PLAUR) is another upregulated MeDEG which is associated with vascular injury in SSc. Iwamoto et al. detected overproduction of PLAUR in vascular smooth muscle cells in SSc [75]. PLAUR induced cell proliferation and suppressed apoptosis of human pulmonary artery smooth muscle cells, resulting in hyperplasia of vascular smooth muscle cells (VSMCs) which is characteristic of proliferative vasculopathy in SSc. The pathogenic role of PLAUR has been supported by detection of elevated level of soluble Urokinase-type Plasminogen Activator receptor in both diffuse and limited cutaneous SSc [76].

ZYX is a novel MeDEG which is upregulated in SSc. A recent publication revealed that Zyxin, a scaffold protein encoded by ZYX, was required in TGF-*β* signaling pathway in response to hypoxia [77]. Nevertheless, the upregulated Zyxin level contributed to cell–matrix adhesion through TGF-*β* signaling [78]. TGF-*β* is known as a pleiotropic cytokine and causes fibroblast activation [79], and ZYX may work in concert with TGF-*β* during the profibrotic process.

Several genes were downregulated and hypermethylated in PBMC of SSc, including F2R, CXCR6, PAG1, PRF1, and RUNX3. Pathway analysis showed that these genes may participate in the activation and proliferation of immune cells. F2R is a gene encoding coagulation factor II (thrombin) receptor, PAR1, which involved in the regulation of thrombotic response. Imbalance of coagulation system followed by autoimmunity has been implicated in the development of fibrosis in lungs [39–41]. PAR1 activation also led to lung vascular leakage. PAR1 immunoreactivity was detected in SSc skin [80]. A recent study showed that thrombin inhibition with dabigatran was protective, thus significantly inhibiting protease-activated receptor-1 (PAR1) activation and the development of pulmonary fibrosis [39]. CXCR6 is the chemokine receptor for angiogenic CXCL16. The increased expression CXCL16 and its receptor in dermal endothelial cells of SSc suggesting CXCL16-CXCR6 may play a role on angiogenesis in SSc skin [37]. PRF1 is a member of granzyme B-Perforin family which is released from NK cells as cytotoxic factor. PRF1 has been reported to be hypermethylated in blood cells of SSc [81] which is in consistent with our result. RUNX3 is a transcription factors participating in the maintenance of genomic stability. Hypomethylated RUNX3 has been reported in dermal fibroblasts of diffuse and limited systemic sclerosis [18].

Other than the aforementioned MeDEGs, most of the DEGs were not associated with their change in DNA methylation and vice versa. As an example, a group of interferon (IFN) signature genes were identified to be significantly upregulated in the DEG profiling, including IFI27, IFI30, IFI35, IFITM3, STAT4, etc. However, no altered methylation sites on these genes were identified. On the other hand, we noticed another set of IFN-inducible genes, PAPP9, MX1, IFI44L, and PLSCR1, which were significantly hypomethylated but no difference on the gene expression level in SSc compared with NC, suggesting the overexpression of IFN-inducible gene was not directly regulated by DNA methylation in PBMC of SSc.

Another group of DEGs with no associated DNA methylation change is NK cell related genes. We found the decreased expression of dozens of genes related to NK cells which implied the impaired innate immunity. Previous studies in peripheral blood were reported with discrepant results. In consistent with our data, defective NK activity was supported by decreased percentage or absolute numbers of NK cells [82, 83], whereas other studies found either normal NK cell counts [84] or increased frequency/number of NK cells in dcSSc with being normal in lcSSc [85]. Our data indicated numbers of genes for NK receptors, KLRs, KIRs, PRF1, and NKTR, may contribute to the reduced NK cell activity. Characteristic KIR genotype, KIR2DS2+/KIR2DL2-, has been associated with SSc [86]. A recent publication suggested the interplay between activating or inhibitory KIR genes with HLA ligands as a critical index of SSc predisposition [87].

In addition to genes showing a reversed correlation between expression and DNA methylation, we also identified four genes, MBP, GNG2, SPTBN1, and NKTR, that were concomitantly downregulated and hypomethylated and one gene, HLA-C, which was concomitantly upregulated and hypermethylated. Apparently regulation of these genes may be governed by other machinery. Prior studies showed that a polymorphic microRNA binding site in the 3'UTR of HLA-C [88]. In fact 24 miRNAs were found differentially expressed in SSc skin [89]. A transcriptional factor Oct1 consensus binding site [90] may account for the differential allele-specific expression level of HLA-C. Transcription of MBP is regulated by heterogeneous nuclear ribonucleoprotein (hnRNP) [91] or alternative splicing [92], indicating distinct regulatory mechanism on gene expression.

The main limitation for this study is that the sample size is relatively small which limited our ability to fully analyze subgroup comparisons within SSc, such as ILD versus non-ILD and dcSSc versus lcSSc. The variation in patient age and disease duration may also impact the interpretation of the results. Future studies should assess methylated gene profile in a large SSc population, which will be selected with more cautiousness on the uniformity of disease onset and duration. The identification of differential expressed gene families in the present study, IFN-related genes and NK cells associated genes, warrants a follow-up investigation in isolated T cells and NK cells.

In conclusion, integration of genome-wide mRNA transcription and DNA methylation profiles distinguished 20 methylation-regulated DEGs. The low overlap frequency between DEGs and DMGs suggested that, for most of the genes in PBMC of SSc, the DNA methylation and gene transcription are two independent molecular events. The identification of MeDEGs in the peripheral circulation of patients suggested a systemic immune response in SSc. Gene network analysis revealed that the 20 MeDEGs comprehensively participate the cascade of immune cell activation, migration, proliferation, and inflammatory response and eventually lead to local pathogenesis. These data indicate that abnormal DNA methylation on susceptibility genes may attribute to the sustained activation of PBMC in peripheral circulation. The gene panel of six MeDEGs by SVM demonstrated high accuracy (100% sensitivity and 100% specificity) in diagnosing SSc. Analysis revealed four under expressed MeDEGs may contribute to the development of ILD. Identification of novel candidate susceptibility genes for SSc may be beneficial in developing biomarkers for diagnostic and prognostic purposes. This study delineated an overall picture regarding epigenetic regulation of autoimmunity in SSc. Our observation laid the groundwork for further diagnostic and mechanistic studies of SSc that could lead to in-depth understanding of the disease.

## Figures and Tables

**Table 1:** Characteristics of clinical subjects and healthy controls.

Clinical characteristics	Controls (*n*=19)	SSc (*n*=18)
Age (Mean ± SD)	39.8 ± 6.5	46.2 ± 12.4
Sex (M/F)	5/14	7/11
Disease duration (months)	NA	48.7 ± 37.3
Organ involvement		
Interstitial lung disease	NA	10
PAH	NA	1
Raynaud's phenomena	NA	16
Gastrointestinal involvement	NA	9
Digital ulcer	NA	5
Serological characteristics		
Anti-Scl-70	NA	12
Anti-RNP	NA	4
Anti-ANA	NA	16
Anti-dsDNA	NA	0
Anti-Jo-1	NA	0
Anti-Sm	NA	0
Anti-SSa	NA	4
Anti-SSb	NA	0
Anti-ANCA	NA	1
Medications		
Prednisone	NA	17
Cyclophosphamide	NA	7

**Table 2:** Differentially expressed gene family in PBMC of SSc patients.

		**Gene family**	**Genes**
Down-regulated	**NK cell associated gene family**	Cluster of differentiation (CD)*∗*	CD2, CD96, CD 160
		Killer cell lectin like receptors, pseudogene	KLRB1, KLRC1, KLRC2, KLRC3, KLRD1, KLRF1, KLRG1
		Killer cell immunoglobulin like receptors	KIR2DL3, KIR2DL5A, KIR2DS1, KIR2DS3, KIR2DS4, KIR3DL1
		Natural cytotoxicity triggering receptor 3	NCR3
		Natural killer cell triggering receptor	NKTR
		Perforin 1	PRF1

Up-regulated	**Interferon-induced genes**	Interferon, alpha-inducible protein 27	IFI27
		Interferon, gamma-inducible protein 30	IFI30
		Interferon induced protein 35	IFI35
		Interferon induced transmembrane protein 3	IFITM3
		Janus kinase 1	JAK1
		Ribonuclease A family member	RNASE I, II, III
		S100 calcium binding protein A11	S100A11
		Signal transducer and activator of transcription 4	STAT4
	**Complement component**	Complement component	C1QA, C1QB, C1QC, C2, C3AR1, C5AR1
	**α** **-defensin**	Defensin *α*	DEFA1, DEFA1B, DEFA3

*∗* These CD molecules are also found in T cells.

**Table:** (a) 12 upregulated MeDEGs identified in SSc

**H** **U** **G** **O** ^**a**^	**HUGO gene ** **n** **a** **m** **e** ^**a**^ **: **function^**b**^	**Function in SSc or related pathology and reference**
C3AR1	Complement C3a Receptor 1: Receptor for the chemotactic and inflammatory peptide anaphylatoxin C3a. This receptor stimulates chemotaxis, granule enzyme release and superoxide anion production.	Inflammatory response. [23]

CSTA	cystatin A: cysteine proteinase inhibitor, encoding Cystatin A, underlies exfoliative ichthyosis	Protease inhibitor in cell-cell adhesion in the basal and lower suprabasal layers. [24]

CTSZ	Cathepsin Z, lysosomal cysteine proteinase and member of the peptidase C1 family: susceptibility locus suggests a role for MC3R and CTSZ in human tuberculosis	Angiogenesis [25, 26]

CTSG	Cathepsin G, granule serine protease	Development of anti-cathepsin G antibodies in sera, function unknown.
		Angiogenesis [27]
		destruction of the lung matrix and acute inflammation propagation [28].

ELANE	Elastase, Neutrophil Expressed: mutations implicated in severe Congenital and cyclic neutropenia	Destruction of extracellular matrix [29, 30]

LTBR	Lymphotoxin Beta Receptor, a member of the tumor necrosis factor receptor superfamily	development of natural killer cells

NFE2	Nuclear Factor, Erythroid 2: Component of the NF-E2 complex essential for regulating the beta-globin control region	Transcription factor

ODF3B	Outer Dense Fiber Of Sperm Tails 3B	Involved in the pathogenic CD4 T cell in MS

PLAUR	Plasminogen Activator, Urokinase Receptor	Coagulation/fibrolytic system [31, 32] Angiogenesis [33, 34]

SAMDC4A	Sterile Alpha Motif Domain Containing 4A: posttranscriptional regulator	No report on SSc or related disease

SPI1	Spi-1 Proto-Oncogene: transcriptional regulator	Transcription factor

ZYX	Zyxin: actin regulator in vascular smooth muscle	Coagulation [35]
		integrity of vasculature [36]

**Table:** (b) 8 downregulated MeDEGs identified in SSc

**H** **U** **G** **O** ^**a**^	**HUGO gene ** **n** **a** **m** **e** ^**a**^ **: function** ^**b**^	**Function in SSc or related pathology and reference**
CXCR6	C-X-C Motif Chemokine Receptor 6, receptor for the C-X-C chemokine CXCL16	Angiogenesis [37, 38]

F2R	Coagulation Factor II Thrombin Receptor	Coagulation/fibrolytic system, fibrosis [39–41]

FYN	Src Family Tyrosine Kinase	Fibrosis [40, 42]

PAG1	Phosphoprotein Membrane Anchor With Glycosphingolipid Microdomains 1	Proliferation of lymphocytes

PRF1	Perforin 1, pore forming protein 1, crucial effector of T and NK cell-mediated cytolysis, highly homologous to complement component C9	cytotoxic activity [43–45] vascular damage [46]

PRKCH	Protein Kinase C Eta, calcium-insensitive, but activated by diacylglycerol (DAG) and phosphatidylserine	Associated in stroke, but no report in SSc or related diseases

RASSF5	Ras Association Domain Family Member 5, Potential tumor suppressor.	No report on SSc or related disease

RUNX3	Runt Related Transcription Factor 3	Transcription factor, induction and suppressive function of Foxp3+ inducible T reg [47]

http://www.genecards.org.

^**a**^Human Genome Organization nomenclature.

^b^Gene function from GeneCards website.

**Table 4:** Molecular interaction networks involved by the 20 MeDEGs.

**ID**	**Molecules in Network**	**Score**	**Focus Molecules**	**Top Diseases and Functions**
**1**	**C3AR1,** Collagen Alpha1, Collagen(s), Cr3, **CTSG**, **CTSZ**, Ecm, **ELANE**, ERK1/2, **F2R,** Fcer1, Fibrinogen, **FYN**, IFN Beta, Ige, IL12 (complex), Immunoglobulin, Integrin, **LTBR**, Mapk, Nfat (family), P38 MAPK, **PAG1**, Par, **PLAUR**, **PRF1**, **PRKCH,** Pro-inflammatory Cytokine, Ras, **RASSF5**, **RUNX3**, **SAMD4A,** SPI1, **SRC**, Vegf	41	15	Cellular Movement, Hematological System Development and Function, Immune Cell Trafficking

**2**	ADCYAP1R1, ADGRL3, Akt, AZU1, caspase, CCL3L1, CD3, CD226, CSDC2, **CXCR6,** ERK, F2RL3, Gpcr, GPR45, GPR63, HISTONE, IGK, Il8r, Interferon alpha, Jnk, KDM5D, LIME1, **NFE2,** NFE2L3, NFkB (complex), P2RY10, PI3K (complex), Pkc(s), **PRKCH**, RNA polymerase II, SIT1, TCR, tubulin, UQCR11, **ZYX**	8	4	Cell-To-Cell Signaling and Interaction, Cell-mediated Immune Response, Cellular Development

**3**	ESR1, **ODF3B,** PKD1	3	1	Connective Tissue Development and Function, Organ Morphology, Skeletal and Muscular System Development and Function

**4**	CASP1, CEP57, CNOT7, **CSTA**, CTSB, CTSH, CTSL, CUL2, CUL4B, DSP, FOXR1, GATA2, GLI2, HERC2, HOXA10, Hsp90, IVL, JUND, KRT1, LOR, MAP2K7, MAP3K1, METTL23, MYOC, PI3, PTN, RAD21, RASSF9, RASSF10, TCF3, TCTN3, UCHL5, USP53, ZDHHC17, ZIC1	2	1	Post-Translational Modification, Dermatological Diseases and Conditions, Hereditary Disorder

## Data Availability

The microarray data for gene expression and methylation has been submitted to the GEO database with accession number GSE117931, https://www.ncbi.nlm.nih.gov/geo/query/acc.cgi?acc=GSE117931.

## Supplementary Materials

Supplementary 1
** Supplementary Figure** **1**: the diseases and functions analysis by integrated pathway analysis using 184 upregulated genes and 269 downregulated genes.** Supplementary Figure** **2**: GO analysis of the downregulated genes (left) and upregulated genes (right) in PBMC from SSc patients as compared to normal controls. Statistically significant GO terms are shown.** Supplementary Figure** **3**: GO analysis of the hypomethylated genes (left) and hypermethylated genes (right) in PBMC from SSc patients as compared to normal controls. Statistically significant GO terms are shown.

Supplementary 2
** Supplementary Table** **1**: the list of primer sequence used in QPCR validation of the 20 MeDEGs.

Supplementary 3
** Supplementary Table** **2**: the complete list of differentially expressed genes (DEGs) identified in PBMC of SSc patients.

Supplementary 4
** Supplementary Table** **3**: the complete list of differentially methylated positions (DMPs) identified in PBMC of SSc patients.

Supplementary 5
** Supplementary Table** **4**: genomic distribution of differentially methylated positions.

Supplementary 6
** Supplementary Table** **5**: the complete list of 20 MeDEGs in PBMC of SSc patients.

## References

[1] Lafyatis R. (2012). Connective tissue disease: SSc-fibrosis takes flight with Wingless inhibition. *Nature Reviews Rheumatology*.

[2] Winstone T. A., Assayag D., Wilcox P. G. (2014). Predictors of mortality and progression in scleroderma-associated interstitial lung disease: A systematic review. *CHEST*.

[3] Ho Y. Y., Lagares D., Tager A. M., Kapoor M. (2014). Fibrosis—a lethal component of systemic sclerosis. *Nature Reviews Rheumatology*.

[4] Chairta P., Nicolaou P., Christodoulou K. (2017). Genomic and genetic studies of systemic sclerosis: A systematic review. *Human Immunology*.

[5] Tsou P.-S., Sawalha A. H. (2017). Unfolding the pathogenesis of scleroderma through genomics and epigenomics. *Journal of Autoimmunity*.

[6] Jin J., Chou Y.-C., Lima M., Zhou D., Zhou X. (2014). Systemic sclerosis is a complex disease associated mainly with immune regulatory and inflammatory genes. *The Open Rheumatology Journal*.

[7] Lei W., Luo Y., Yan K. (2009). Abnormal DNA methylation in CD4^+^ T cells from patients with systemic lupus erythematosus, systemic sclerosis, and dermatomyositis. *Scandinavian Journal of Rheumatology*.

[8] Lian X., Xiao R., Hu X. (2012). DNA demethylation of CD40L in CD4+ T cells from women with systemic sclerosis: A possible explanation for female susceptibility. *Arthritis & Rheumatology*.

[9] Rosato E., Letizia C., Proietti M. (2009). Plasma adrenomedullin and endothelin-1 levels are reduced and Raynaud's phenomenon improved by daily tadalafil administration in male patients with Systemic Sclerosis. *Journal of Biological Regulators and Homeostatic Agents*.

[10] Broen J. C. A., Radstake T. R. D. J., Rossato M. (2014). The role of genetics and epigenetics in the pathogenesis of systemic sclerosis. *Nature Reviews Rheumatology*.

[11] Bjornsson H. T., Fallin M. D., Feinberg A. P. (2004). An integrated epigenetic and genetic approach to common human disease. *Trends in Genetics*.

[12] Jaenisch R., Bird A. (2003). Epigenetic regulation of gene expression: how the genome integrates intrinsic and environmental signals. *Nature Genetics*.

[13] Altorok N., Tsou P.-S., Coit P., Khanna D., Sawalha A. H. (2015). Genome-wide DNA methylation analysis in dermal fibroblasts from patients with diffuse and limited systemic sclerosis reveals common and subset-specific DNA methylation aberrancies. *Annals of the Rheumatic Diseases*.

[14] Wang Y., Fan P.-S., Kahaleh B. (2006). Association between enhanced type I collagen expression and epigenetic repression of the FLI1 gene in scleroderma fibroblasts. *Arthritis & Rheumatology*.

[15] Wang Y., Shu Y., Xiao Y. (2014). Hypomethylation and overexpression of ITGAL (CD11a) in CD4+ T cells in systemic sclerosis. *Clinical Epigenetics*.

[16] Matatiele P., Tikly M., Tarr G., Gulumian M. (2015). DNA methylation similarities in genes of Black South Africans with Systemic lupus erythematosus and Systemic sclerosis. *Journal of Biomedical Science*.

[17] Wang Y., Kahaleh B. (2013). Epigenetic repression of bone morphogenetic protein receptor II expression in scleroderma. *Journal of Cellular and Molecular Medicine*.

[18] Van Den Hoogen F., Khanna D., Fransen J. (2014). OP0033Classification Criteria for Systemic Sclerosis: Preliminary Results. *Annals of the Rheumatic Diseases*.

[19] Kazarooni E. A., Martinez F. J., Flint A. (1997). Thin-section CT obtained at 10-mm increments versus limited three-level thin-section CT for idiopathic pulmonary fibrosis: correlation with pathologic scoring. *American Journal of Roentgenology*.

[20] Orens J. B., Kazerooni E. A., Martinez F. J. (1995). The sensitivity of high-resolution CT in detecting idiopathic pulmonary fibrosis proved by open lung biopsy: A prospective study. *CHEST*.

[21] American Thoracic Society (ATS) (2000). Idiopathic pulmonary fibrosis: diagnosis and treatment. International consensus statement. *American Journal of Respiratory and Critical Care Medicine*.

[22] Denton C. P., Khanna D. (2017). Systemic sclerosis. *The Lancet*.

[23] Kemper C., Atkinson J. P. (2007). T-cell regulation: With complements from innate immunity. *Nature Reviews Immunology*.

[24] Blaydon D. C., Nitoiu D., Eckl K.-M. (2011). Mutations in CSTA, encoding cystatin A, underlie exfoliative ichthyosis and reveal a role for this protease inhibitor in cell-cell adhesion. *American Journal of Human Genetics*.

[25] Kang M.-L., Kim J.-E., Im G.-I. (2017). Vascular endothelial growth factor-transfected adipose-derived stromal cells enhance bone regeneration and neovascularization from bone marrow stromal cells. *Journal of Tissue Engineering and Regenerative Medicine*.

[26] Buhler A., Berger S., Bengsch F. (2013). Cathepsin proteases promote angiogenic sprouting and laser-induced choroidal neovascularisation in mice. *Experimental Eye Research*.

[27] Wilson T. J., Nannuru K. C., Futakuchi M., Singh R. K. (2010). Cathepsin G-mediated enhanced TGF-beta signaling promotes angiogenesis via upregulation of VEGF and MCP-1. *Cancer Letters*.

[28] Craciun I., Fenner A. M., Kerns R. J. (2016). N-Arylacyl O-sulfonated aminoglycosides as novel inhibitors of human neutrophil elastase, cathepsin G and proteinase 3. *Glycobiology*.

[29] Kristensen J. H., Karsdal M. A., Sand J. M. B. (2015). Serological assessment of neutrophil elastase activity on elastin during lung ECM remodeling. *BMC Pulmonary Medicine*.

[30] Hara T., Ogawa F., Yanaba K. (2009). Elevated serum concentrations of polymorphonuclear neutrophilic leukocyte elastase in systemic sclerosis: association with pulmonary fibrosis. *The Journal of Rheumatology*.

[31] Mahoney J. M., Taroni J., Martyanov V. (2015). Systems Level Analysis of Systemic Sclerosis Shows a Network of Immune and Profibrotic Pathways Connected with Genetic Polymorphisms. *PLoS Computational Biology*.

[32] Manetti M., Allanore Y., Revillod L. (2011). A genetic variation located in the promoter region of the UPAR (CD87) gene is associated with the vascular complications of systemic sclerosis. *Arthritis & Rheumatology*.

[33] D'Alessio S., Fibbi G., Cinelli M. (2004). Matrix metalloproteinase 12-dependent cleavage of urokinase receptor in systemic sclerosis microvascular endothelial cells results in impaired angiogenesis. *Arthritis & Rheumatology*.

[34] Serrati S., Cinelli M., Margheri F. (2006). Systemic sclerosis fibroblast inhibit in vitro angiogenesis by MMP-12-dependent cleavage of the endothelial cell urokinase receptor. *The Journal of Pathology*.

[35] Han X., Li P., Yang Z. (2017). Zyxin regulates endothelial von Willebrand factor secretion by reorganizing actin filaments around exocytic granules. *Nature Communications*.

[36] Saphirstein R. J., Gao Y. Z., Lin Q. Q., Morgan K. G. (2015). Cortical actin regulation modulates vascular contractility and compliance in veins. *The Journal of Physiology*.

[37] Rabquer B. J., Tsou P.-S., Hou Y. (2011). Dysregulated expression of MIG/CXCL9, IP-10/CXCL10 and CXCL16 and their receptors in systemic sclerosis. *Arthritis Research & Therapy*.

[38] Isozaki T., Arbab A. S., Haas C. S. (2013). Evidence that CXCL16 is a potent mediator of angiogenesis and is involved in endothelial progenitor cell chemotaxis : studies in mice with K/BxN serum-induced arthritis. *Arthritis & Rheumatology*.

[39] Shea B. S., Probst C. K., Brazee P. L. (2017). Uncoupling of the profibrotic and hemostatic effects of thrombin in lung fibrosis. *JCI Insight*.

[40] Grove L. M., Southern B. D., Jin T. H. (2014). Urokinase-type plasminogen activator receptor (upar) ligation induces a raft-localized integrin signaling switch that mediates the hypermotile phenotype of fibrotic fibroblasts. *The Journal of Biological Chemistry*.

[41] Scotton C. J., Krupiczojc M. A., Königshoff M. (2009). Increased local expression of coagulation factor X contributes to the fibrotic response in human and murine lung injury. *The Journal of Clinical Investigation*.

[42] Vepachedu R., Gorska M. M., Singhania N., Cosgrove G. P., Brown K. K., Alam R. (2007). Unc119 regulates myofibroblast differentiation through the activation of Fyn and the p38 MAPK pathway. *The Journal of Immunology*.

[43] Henriques A., Silva C., Santiago M. (2016). Subset-specific alterations in frequencies and functional signatures of gammadelta T cells in systemic sclerosis patients. *Inflammation Research : Official Journal of the European Histamine Research Society*.

[44] Gilani S. R., Vuga L. J., Lindell K. O. (2010). CD28 down-regulation on circulating CD4 T-cells is associated with poor prognoses of patients with idiopathic pulmonary fibrosis. *PLoS ONE*.

[45] Miyazaki H., Kuwano K., Yoshida K. (2004). The perforin mediated apoptotic pathway in lung injury and fibrosis. *Journal of Clinical Pathology*.

[46] Choy J. C., Kerjner A., Wong B. W., McManus B. M., Granville D. J. (2004). Perforin mediates endothelial cell death and resultant transplant vascular disease in cardiac allografts. *The American Journal of Pathology*.

[47] Klunker S., Chong M. M. W., Mantel P.-Y. (2009). Transcription factors RUNX1 and RUNX3 in the induction and suppressive function of Foxp3+ inducible regulatory T cells. *The Journal of Experimental Medicine*.

[48] Du P., Kibbe W. A., Lin S. M. (2008). *lumi*: a pipeline for processing Illumina microarray. *Bioinformatics*.

[49] Barbosa-Morais N. L., Dunning M. J., Samarajiwa S. A. (2009). A re-annotation pipeline for Illumina BeadArrays: Improving the interpretation of gene expression data. *Nucleic Acids Research*.

[50] Storey J. D., Tibshirani R. (2003). Statistical significance for genomewide studies. *Proceedings of the National Acadamy of Sciences of the United States of America*.

[51] Morris T. J., Butcher L. M., Feber A. (2014). ChAMP: 450k chip analysis methylation pipeline. *Bioinformatics*.

[52] Noble W. S. (2006). What is a support vector machine?. *Nature Biotechnology*.

[53] Furey T., Cristianini N., Duffy N., Bednarski D. W., Schummer M., Haussler D. (2000). Support vector machine classification and validation of cancer tissue samples using microarray expression data. *Bioinformatics*.

[54] Huang D. W., Sherman B. T., Lempicki R. A. (2009). Systematic and integrative analysis of large gene lists using DAVID bioinformatics resources. *Nature Protocols*.

[55] Luo W., Friedman M. S., Shedden K., Hankenson K. D., Woolf P. J. (2009). GAGE: generally applicable gene set enrichment for pathway analysis. *BMC Bioinformatics*.

[56] Luo W., Brouwer C. (2013). Pathview: An R/Bioconductor package for pathway-based data integration and visualization. *Bioinformatics*.

[57] Hannon E., Spiers H., Viana J. (2015). Methylation QTLs in the developing brain and their enrichment in schizophrenia risk loci. *Nature Neuroscience*.

[58] Tan F. K., Zhou X., Mayes M. D. (2006). Signatures of differentially regulated interferon gene expression and vasculotrophism in the peripheral blood cells of systemic sclerosis patients. *Rheumatology*.

[59] Brkic Z., Van Bon L., Cossu M. (2016). The interferon type i signature is present in systemic sclerosis before overt fibrosis and might contribute to its pathogenesis through high BAFF gene expression and high collagen synthesis. *Annals of the Rheumatic Diseases*.

[60] Okrój M., Johansson M., Saxne T., Blom A. M., Hesselstrand R. (2016). Analysis of complement biomarkers in systemic sclerosis indicates a distinct pattern in scleroderma renal crisis. *Arthritis Research & Therapy*.

[61] Scambi C., Ugolini S., Sakari Jokiranta T. (2015). The local complement activation on vascular bed of patients with systemic sclerosis: A hypothesis-generating study. *PLoS ONE*.

[62] Jarczak J., Kościuczuk E. M., Lisowski P. (2013). Defensins: natural component of human innate immunity. *Human Immunology*.

[63] Sakamoto N., Kakugawa T., Hara A. (2015). Association of elevated *α*-defensin levels with interstitial pneumonia in patients with systemic sclerosis. *Respiratory Research*.

[64] Imgenberg-Kreuz J., Sandling J. K., Almlöf J. C. (2016). Genome-wide DNA methylation analysis in multiple tissues in primary Sjögren's syndrome reveals regulatory effects at interferon-induced genes. *Annals of the Rheumatic Diseases*.

[65] Kato S., Kishiro I., Ohnuma N. (2000). Suppressive effect of saprogrelate hydrochloride on Raynaud's phenomenon and respiratory failure in patients with systemic sclerosis. *Respirology*.

[66] Kowal-Bielecka O., Kowal K., Lewszuk A., Bodzenta-Lukaszyk A., Walecki J., Sierakowski S. (2005). *β* thromboglobulin and platelet factor 4 in bronchoalveolar lavage fluid of patients with systemic sclerosis. *Annals of the Rheumatic Diseases*.

[67] Macko R. F., Gelber A. C., Young B. A. (2002). Increased circulating concentrations of the counteradhesive proteins SPARC and thrombospondin-1 in systemic sclerosis (scleroderma). Relationship to platelet and endothelial cell activation. *The Journal of Rheumatology*.

[68] Tozkır J. D., Tozkır H., Gürkan H. (2016). The investigation of killer cell immunoglobulin-like receptor genotyping in patients with systemic lupus erytematosus and systemic sclerosis. *Clinical Rheumatology*.

[69] Rauch T. A., Wu X., Zhong X., Riggs A. D., Pfeifer G. P. (2009). A human B cell methylome at 100-base pair resolution. *Proceedings of the National Acadamy of Sciences of the United States of America*.

[70] Ball M. P., Li J. B., Gao Y. (2009). Targeted and genome-scale strategies reveal gene-body methylation signatures in human cells. *Nature Biotechnology*.

[71] Zhu H., Mi W., Luo H. (2016). Whole-genome transcription and DNA methylation analysis of peripheral blood mononuclear cells identified aberrant gene regulation pathways in systemic lupus erythematosus. *Arthritis Research & Therapy*.

[72] Tsou P.-S., Wren J. D., Amin M. A. (2016). Histone deacetylase 5 is overexpressed in scleroderma endothelial cells and impairs angiogenesis via repression of proangiogenic factors. *Arthritis & Rheumatology*.

[73] Wagner C. J., Schultz C., Mall M. A. (2016). Neutrophil elastase and matrix metalloproteinase 12 in cystic fibrosis lung disease. *Molecular and Cellular Pediatrics*.

[74] Akkari L., Gocheva V., Kester J. C. (2014). Distinct functions of macrophage-derived and cancer cell-derived cathepsin Z combine to promote tumor malignancy via interactions with the extracellular matrix. *Genes & Development*.

[75] Iwamoto N., Vettori S., Maurer B. (2016). Downregulation of miR-193b in systemic sclerosis regulates the proliferative vasculopathy by urokinase-type plasminogen activator expression. *Annals of the Rheumatic Diseases*.

[76] Legány N., Toldi G., Distler J. H. W. (2015). Increased plasma soluble urokinase plasminogen activator receptor levels in systemic sclerosis: Possible association with microvascular abnormalities and extent of fibrosis. *Clinical Chemistry and Laboratory Medicine*.

[77] Ma B., Cheng H., Gao R. (2016). Zyxin-Siah2-Lats2 axis mediates cooperation between Hippo and TGF-*β* signalling pathways. *Nature Communications*.

[78] Bianchi-Smiraglia A., Kunnev D., Limoge M., Lee A., Beckerle M. C., Bakin A. V. (2013). Integrin-*β*5 and zyxin mediate formation of ventral stress fibers in response to transforming growth factor *β*. *Cell Cycle*.

[79] Asano Y., Ihn H., Yamane K., Jinnin M., Mimura Y., Tamaki K. (2005). Increased expression of integrin *α*v*β*3 contributes to the establishment of autocrine TGF-*β* signaling in scleroderma fibroblasts. *The Journal of Immunology*.

[80] Cevikbas F., Seeliger S., Fastrich M. (2011). Role of protease-activated receptors in human skin fibrosis and scleroderma. *Experimental Dermatology*.

[81] Mercer P. F., Deng X., Chambers R. C. (2007). Signaling pathways involved in proteinase-activated receptor1-induced proinflammatory and profibrotic mediator release following lung injury. *Annals of the New York Academy of Sciences*.

[82] Riccieri V., Parisi G., Spadaro A. (2005). Reduced circulating natural killer T cells and gamma/delta T cells in patients with systemic sclerosis. *The Journal of Rheumatology*.

[83] Parolin Ercole L., Malvezzi M., Boaretti A. C., Ramos da Rosa Utiyama S., Rachid A. (2003). Analysis of lymphocyte subpopulations in systemic sclerosis. *Journal of Investigational Allergology and Clinical Immunology*.

[84] Gambichler T., Tigges C., Burkert B., Hoxtermann S., Altmeyer P., Kreuter A. (2010). Absolute count of T and B lymphocyte subsets is decreased in systemic sclerosis. *European Journal of Medical Research*.

[85] Horikawa M., Hasegawa M., Komura K. (2005). Abnormal natural killer cell function in systemic sclerosis: altered cytokine production and defective killing activity. *Journal of Investigative Dermatology*.

[86] Momot T., Koch S., Hunzelmann N. (2004). Association of Killer Cell Immunoglobulin-Like Receptors with Scleroderma. *Arthritis & Rheumatology*.

[87] Mahmoudi M., Fallahian F., Sobhani S. (2017). Analysis of killer cell immunoglobulin-like receptors (KIRs) and their HLA ligand genes polymorphisms in Iranian patients with systemic sclerosis. *Clinical Rheumatology*.

[88] Kulkarni S., Qi Y., O'hUigin C. (2013). Genetic interplay between HLA-C and MIR148A in HIV control and Crohn disease. *Proceedings of the National Acadamy of Sciences of the United States of America*.

[89] Li H., Yang R., Fan X. (2012). MicroRNA array analysis of microRNAs related to systemic scleroderma. *Rheumatology International*.

[90] Vince N., Li H., Ramsuran V. (2016). HLA-C Level Is Regulated by a Polymorphic Oct1 Binding Site in the HLA-C Promoter Region. *American Journal of Human Genetics*.

[91] White R., Gonsior C., Bauer N. M., Krämer-Albers E.-M., Luhmann H. J., Trotter J. (2012). Heterogeneous nuclear ribonucleoprotein (hnRNP) F is a novel component of oligodendroglial RNA transport granules contributing to regulation of myelin basic protein (MBP) synthesis. *The Journal of Biological Chemistry*.

[92] Kamholz J., Toffenetti J., Lazzarini R. A. (1988). Organization and expression of the human myelin basic protein gene. *Journal of Neuroscience Research*.

