# Supplementary material for: Integration of Genome-Wide DNA Methylation and Transcription Uncovered Aberrant Methylation-Regulated Genes and Pathways in the Peripheral Blood Mononuclear Cells of Systemic Sclerosis
Source: Int J Rheumatol. 2018 Sep 2;2018:7342472. doi: 10.1155/2018/7342472 (PMC6139224; doi:10.1155/2018/7342472)
Supplement: Supplementary 1 — Supplementary Figure 1: the diseases and functions analysis by integrated pathway analysis using 184 upregulated genes and 269 downregulated genes. Supplementary Figure 2: GO analysis of the downregulated genes (left) and upregulated genes (right) in PBMC from SSc patients as compared to normal controls. Statistically significant GO terms are shown. Supplementary Figure 3: GO analysis of the hypomethylated genes (left) and hypermethylated genes (right) in PBMC from SSc patients as compared to normal controls. Statistically significant GO terms are shown. [file 7342472.f1.pptx]

## Slide 1
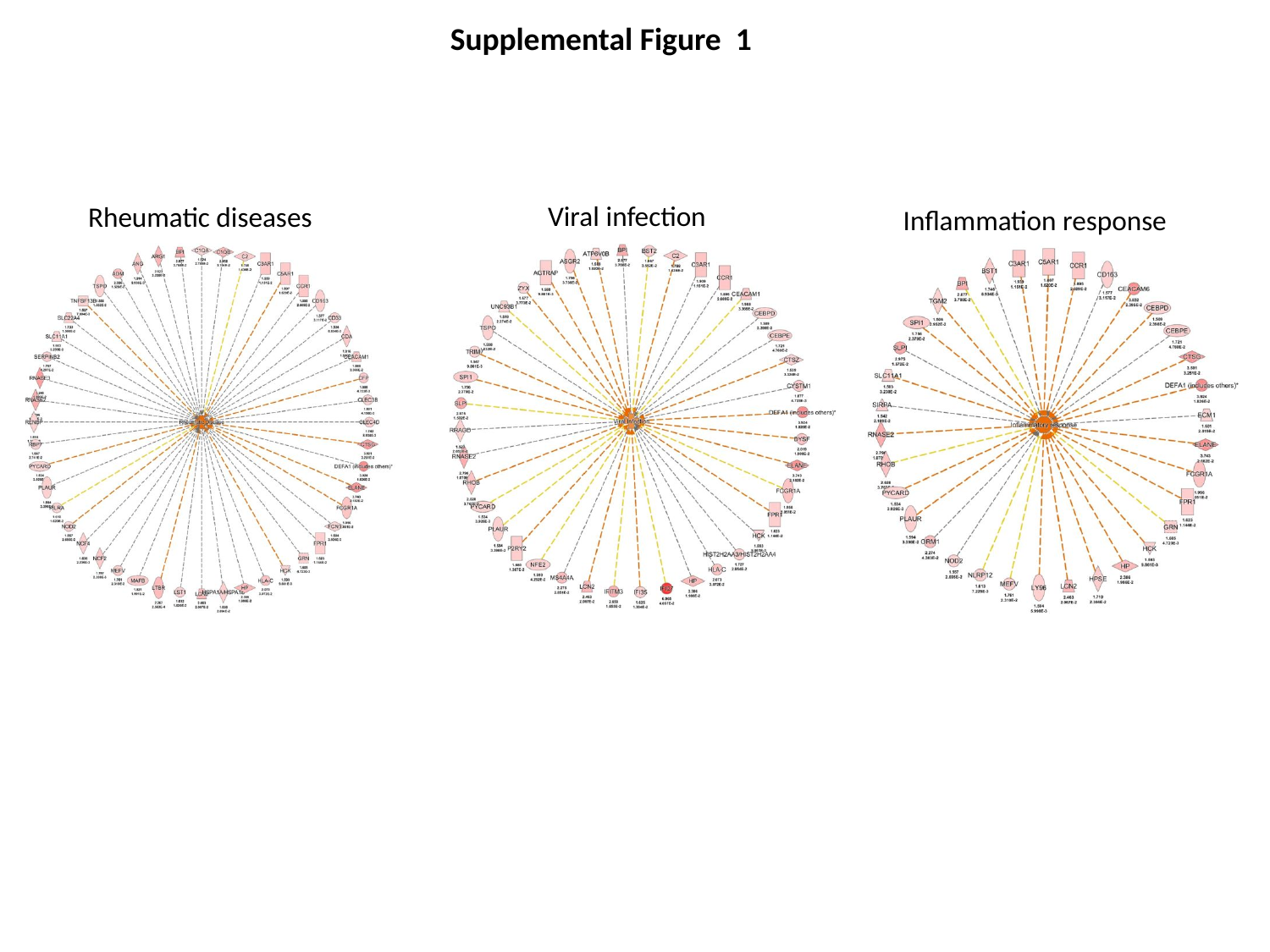

Supplemental Figure 1
Viral infection
Rheumatic diseases
Inflammation response

## Slide 2
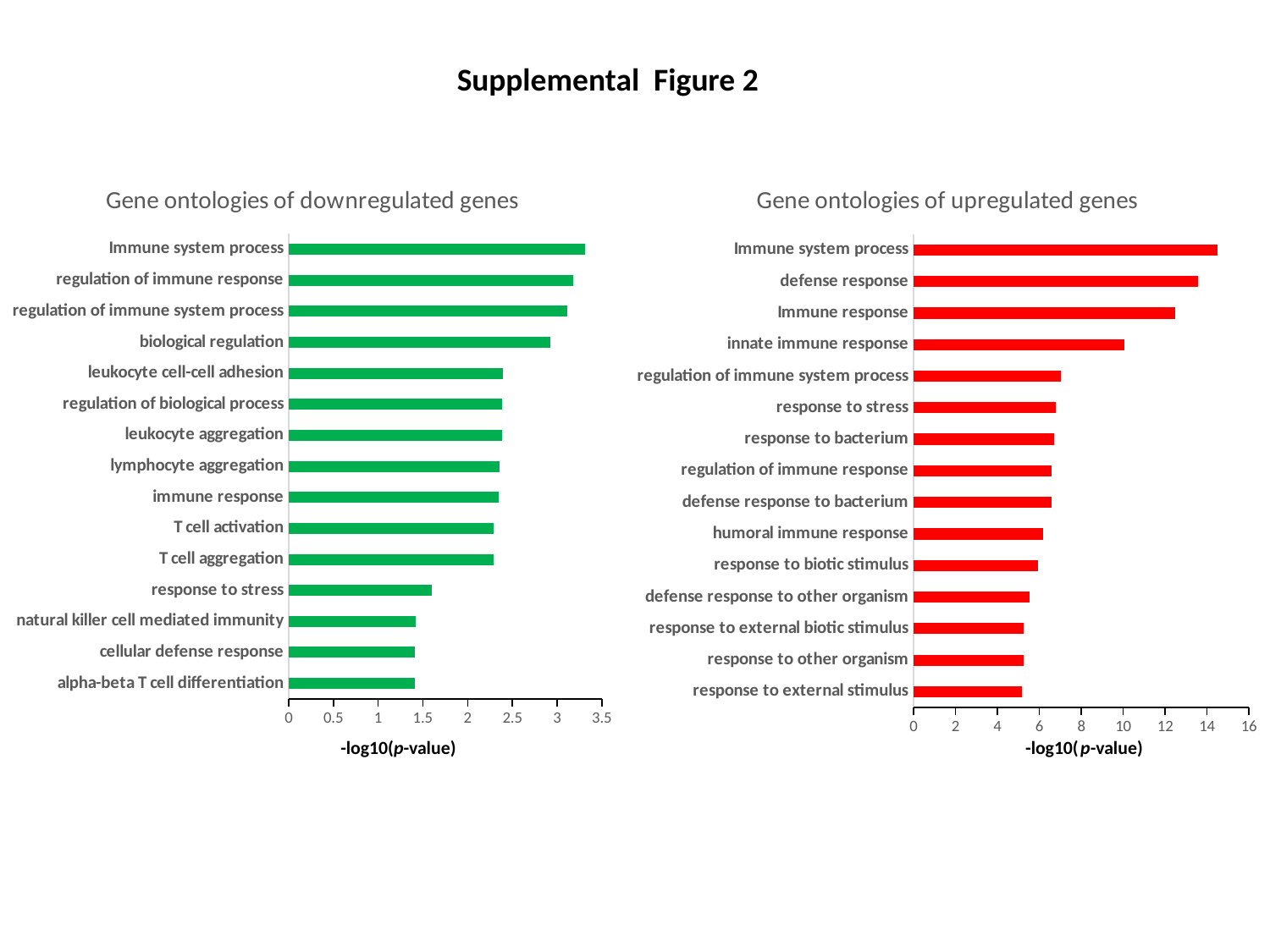

Supplemental Figure 2
### Chart: Gene ontologies of downregulated genes
| Category | |
|---|---|
| alpha-beta T cell differentiation | 1.408935392973501 |
| cellular defense response | 1.408935392973501 |
| natural killer cell mediated immunity | 1.4202164033831899 |
| response to stress | 1.6020599913279623 |
| T cell aggregation | 2.2924298239020637 |
| T cell activation | 2.2924298239020637 |
| immune response | 2.3467874862246565 |
| lymphocyte aggregation | 2.3565473235138126 |
| leukocyte aggregation | 2.3872161432802645 |
| regulation of biological process | 2.3872161432802645 |
| leukocyte cell-cell adhesion | 2.3979400086720375 |
| biological regulation | 2.9208187539523753 |
| regulation of immune system process | 3.113509274827518 |
| regulation of immune response | 3.1804560644581312 |
| Immune system process | 3.309803919971486 |
### Chart: Gene ontologies of upregulated genes
| Category | |
|---|---|
| response to external stimulus | 5.187086643357144 |
| response to other organism | 5.2441251443275085 |
| response to external biotic stimulus | 5.2441251443275085 |
| defense response to other organism | 5.522878745280337 |
| response to biotic stimulus | 5.920818753952375 |
| humoral immune response | 6.167491087293763 |
| defense response to bacterium | 6.568636235841013 |
| regulation of immune response | 6.585026652029182 |
| response to bacterium | 6.698970004336019 |
| response to stress | 6.795880017344075 |
| regulation of immune system process | 7.008773924307505 |
| innate immune response | 10.065501548756432 |
| Immune response | 12.481486060122112 |
| defense response | 13.568636235841012 |
| Immune system process | 14.508638306165727 |-log10(p-value) -log10(p-value)

## Slide 3
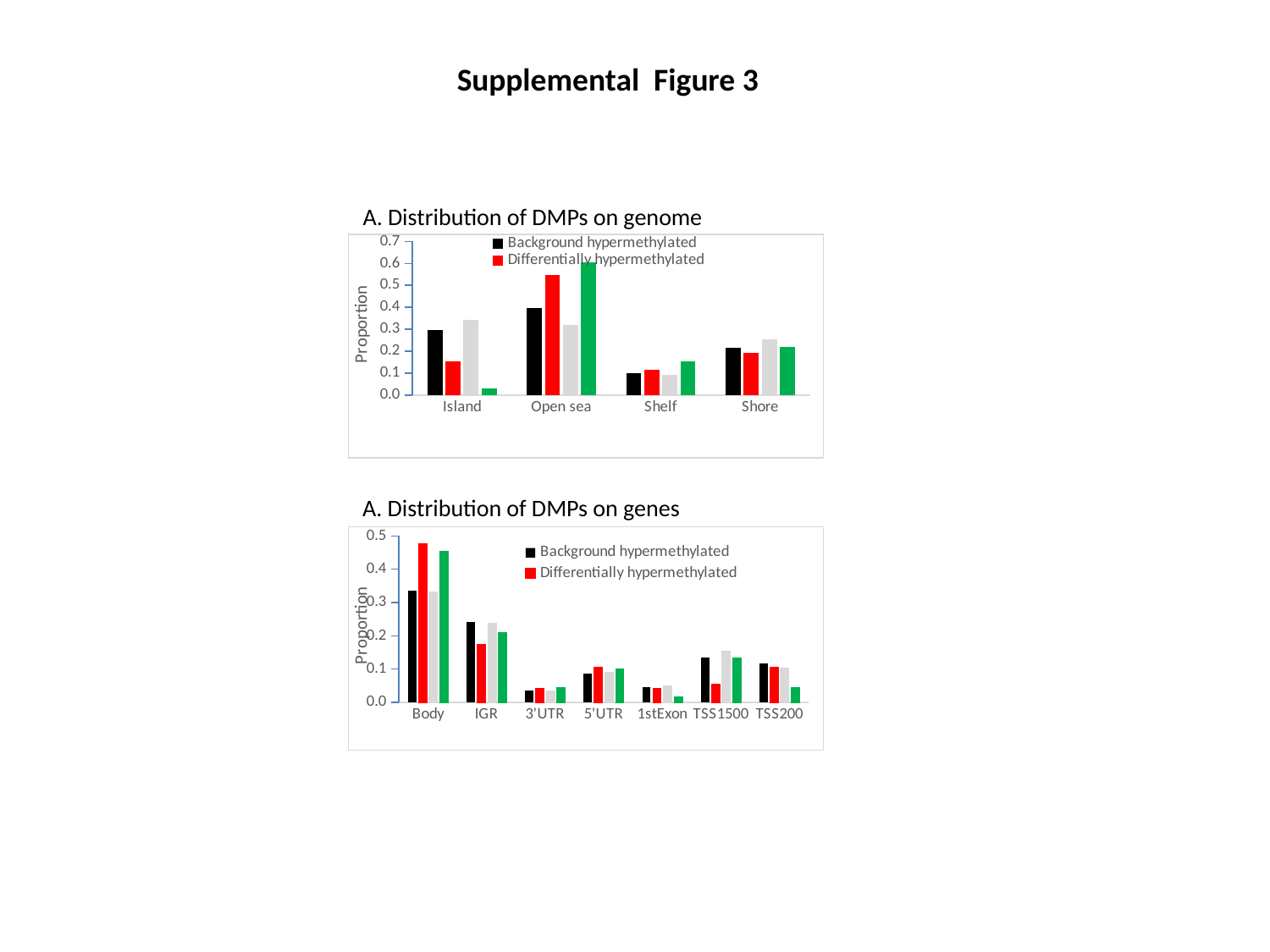

Supplemental Figure 3
A. Distribution of DMPs on genome
### Chart
| Category | | | | |
|---|---|---|---|---|
| Island | 0.29434285 | 0.15384615384615385 | 0.34279789 | 0.029411764705882353 |
| Open sea | 0.39368044 | 0.5454545454545454 | 0.31605696 | 0.6023017902813299 |
| Shelf | 0.09883802 | 0.11188811188811189 | 0.08875241 | 0.15089514066496162 |
| Shore | 0.21313869 | 0.1888111888111888 | 0.25239273 | 0.21739130434782608 |A. Distribution of DMPs on genes
### Chart
| Category | | | | |
|---|---|---|---|---|
| Body | 0.33643643 | 0.4755244755244755 | 0.33056841 | 0.45268542199488493 |
| IGR | 0.2418976 | 0.17482517482517482 | 0.2368533 | 0.20971867007672634 |
| 3'UTR | 0.03582494 | 0.04195804195804196 | 0.03533702 | 0.043478260869565216 |
| 5'UTR | 0.08678589 | 0.1048951048951049 | 0.09127364 | 0.1010230179028133 |
| 1stExon | 0.04667618 | 0.04195804195804196 | 0.04899328 | 0.015345268542199489 |
| TSS1500 | 0.13477344 | 0.055944055944055944 | 0.1528376 | 0.13427109974424553 |
| TSS200 | 0.11760552 | 0.1048951048951049 | 0.10413676 | 0.043478260869565216 |

## Slide 4
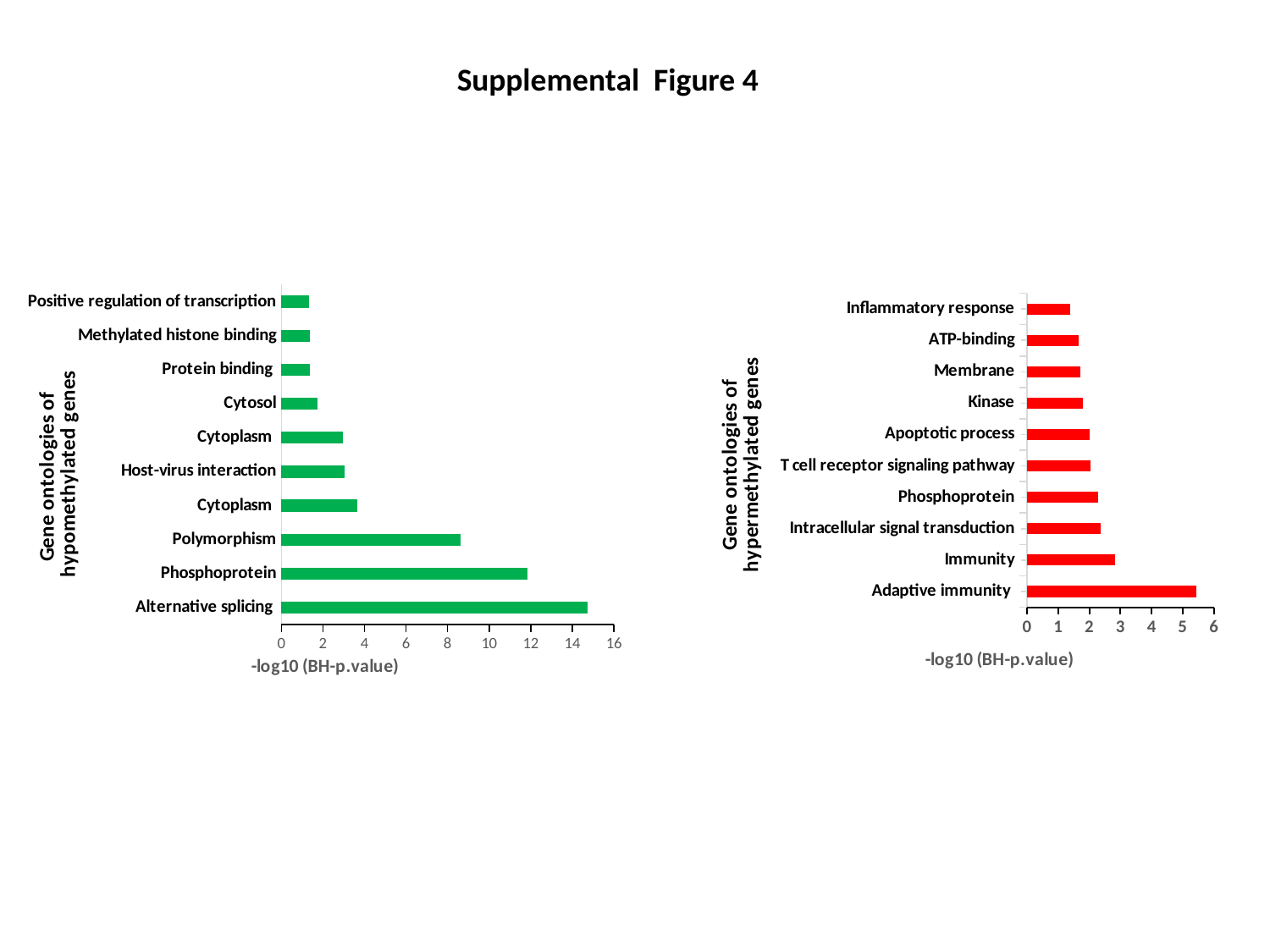

Supplemental Figure 4
### Chart
| Category | |
|---|---|
| Alternative splicing | 14.721246399047171 |
| Phosphoprotein | 11.853871964321762 |
| Polymorphism | 8.638272163982407 |
| Cytoplasm | 3.657577319177794 |
| Host-virus interaction | 3.050609993355087 |
| Cytoplasm | 2.958607314841775 |
| Cytosol | 1.7212463990471711 |
| Protein binding | 1.3665315444204136 |
| Methylated histone binding | 1.3565473235138126 |
| Positive regulation of transcription | 1.337242168318426 |
### Chart
| Category | |
|---|---|
| Adaptive immunity | 5.431798275933005 |
| Immunity | 2.8239087409443187 |
| Intracellular signal transduction | 2.3565473235138126 |
| Phosphoprotein | 2.2924298239020637 |
| T cell receptor signaling pathway | 2.0268721464003012 |
| Apoptotic process | 2.0 |
| Kinase | 1.7958800173440752 |
| Membrane | 1.7212463990471711 |
| ATP-binding | 1.6575773191777938 |
| Inflammatory response | 1.3872161432802645 |
